# Editorial: Rumen microbiome dynamics and their implications in health and environment

**DOI:** 10.3389/fmicb.2023.1223885

**Published:** 2023-06-06

**Authors:** Robert W. Li, Christina D. Moon, Diego P. Morgavi

**Affiliations:** ^1^United States Department of Agriculture, Agricultural Research Service (USDA-ARS), Beltsville, MD, United States; ^2^AgResearch Ltd., Grassland Research Centre, Palmerston North, New Zealand; ^3^INRAE, Clermont-Auvergne-Rhône-Alpes, Saint-Genès-Champanelle, France

**Keywords:** rumen, microbial ecosystem, resilience, methane, ruminant (source: MeSH), inhibitor, early life

As part of the unique digestive system of ruminants, the rumen harbors a dazzling array of microbial diversity. As members of a complex and dynamic microbial ecosystem, bacteria, protozoa, fungi, archaea and viruses in the rumen interact with each other and contribute, individually or in concert, to rumen function. Ruminal microorganisms convert plant fiber into short-chain fatty acids (SCFA) and other metabolites for the production of meat and milk, a key determinant for feed use efficiency. Rumen microbes are also capable of detoxification and metabolizing xenobiotics, particularly aromatic compounds, and secondary plant metabolites. As a result, the rumen as an efficient bioremediation system can be used to clean up environmental toxins. Ruminal biohydrogenation, a process by which bacteria convert dietary unsaturated fatty acids to saturated fatty acids, affects fatty acid profiles of ruminant products. On the other hand, rumen methanogens are a major contributor to methane emissions; ruminant farming accounts for up to 25% of global anthropogenic methane emissions. For example, a cow can release up to 500 L of methane each day; globally, cattle production releases up to 100 million tons of methane annually. Moreover, methane is 28 times more potent than CO_2_ in contributing to global warming. Dozens of methane inhibitors have been tested in the past decades. Although some of these inhibitors reduce methane production by up to 90%, obstacles prevent their widespread use. Inhibition may be transient, lasting only a few weeks and dissipates when inhibitors are withdrawn. When used at doses required to achieve strong effects, many methane inhibitors interfere with feed intake, digestion, and rumen fermentation, harming production traits such as live weight gain. Interventions that incur costs and losses to producers hamper sustainable technology transfer.

The collective efforts of a total of 97 authors from 16 countries, from Austria to the United States of America, resulted in the publication of 15 papers in the Research Topic entitled “Rumen microbiome dynamics and their implications in health and environment.” This special collection covers a broad range of research subjects related to the rumen microbiome and its manipulation. The host species examined include multiple breeds of major ruminant species, sheep, goats, and cattle, under various production systems.

First, Lobo and Faciola conducted a comprehensive review of the recent advances in ruminal viruses that infect not only bacteria (bacteriophages or phages), but also archaea (archaeaphages) and the eukaryotes fungi (mycophages) and protozoa as well as their role in modulating the interactions among other ruminal microorganisms. These viruses, resident or transient in the rumen environment, are concentrated in a handful of viral families and associated with the dominant ruminal bacterial phyla. The authors also discussed the potential application of ruminal phage therapy in livestock production, including in controlling pathogens and microbes harboring antibiotic resistant genes, reducing methane production, and regulating gut homeostasis, such as reverting rumen dysbiosis and alleviating ruminal acidosis.

Five publications in this collection investigated the establishment and development of the rumen microbiota during early life in three major ruminant species, sheep, goats and cattle. Mao et al. compared two weaning schemes, 30-day weaning and 45-day weaning, as well as their respective 5-days post-weaning controls in male Hu lambs, on the rumen bacterial and archaeal colonization and development. While the weaning schemes have little effect on the rumen archaeal population, the rumen bacterial communities became more stable and experienced less fluctuation at the later weaning age, suggesting that later weaning may benefit the rumen microbial establishment. Further, several taxa, such as *Fibrobacter*, had a significant and positive correlation with rumen papillae length. In another study (Yin et al.), the rumen bacterial establishment in the same sheep breed was monitored from birth to 120 days of age. The authors concluded that the rumen microbial community and function of rumen, as judged by its lack of resilience and resistance to disturbances, are not well-established before lambs reach 20 days of age, providing a window of opportunities for ruminal intervention for desired growth performance at later stages. Artiles-Ortega et al. attempted to understand if prenatal exposure to a protein-rich legume *Leucaena leucocephala* with toxic secondary metabolites would affect post-weaning performance and immune status in goat kids. Their findings suggest that the prenatal adaptation increases dry matter intake after weaning, resulting in higher daily bodyweight gain. Furthermore, postnatal supplementation of live yeast favors the maturation of the rumen bacterial community and protozoa colonization; and early-life dietary interventions can have persistent effects on production traits. In calves, Huuki et al. demonstrated that rumen fluid from an adult cow administered fresh to calves for 6 weeks prior to weaning enhanced the maturation of rumen bacterial and archaeal communities and resulted in improved feed intake and significant weight gain compared to untreated monozygotic twin calves used as controls. Cristobal-Carballo et al. investigated the effect of divergent feeding regimes during the first 41 weeks of life of Hereford–Friesian-cross female calves on long-term rumen fermentation performance and found that the rumen microbiota and associated fermentation end-products are largely driven by the diet consumed at the time of sampling and that early dietary interventions have little detectable long-term microbial imprint potential on rumen function.

Beef production relying on the use of resilient (rustic) breeds may alleviate some of the challenges posed by climate change, due to their ability for better adaptation and efficient utilization of low-quality fibers. Daghio et al. compared the rumen microbial composition and metabolites as well as growth traits of two rustic breeds under two production systems, feedlot and pasture grazing. Their findings suggest that while the production system shapes the rumen microbiome structure, the cattle breed is the main factor that influences bacterial communities, supporting the notion that host genetics may play an important role in determining rumen microbial composition. Furthermore, steer performance is likely affected by the rumen capacity for SCFA production and the presence of hydrogen sinks that divert hydrogen to processes alternative to methanogenesis. In dairy cattle, progressive mechanisms of adaptation in the rumen and hindgut of cows fed increasing amounts of starch with or without a phytogenic feed additive have been investigated by Ricci et al.. The gradual inclusion of starch in the diet and the phytogenic additive altered microbiota composition and metabolic activity in all gut compartments surveyed. Further, the microbiota has capacity to adjust differentially, yet rapidly, to changing dietary conditions. Last, Li et al. examined the effect of herbal tea residue on the growth performance of Simmental crossbred finishing steers. Their findings demonstrate that while herbal tea residue does not appear to promote cattle growth, its supplementation tend to improve rumen fermentation by increasing the propionic acid concentration and the propionate-to-acetate ratio, indirectly leading to the improvement of muscle quality, such as tenderness and oleic acid and linoleic acid concentrations in the longissimus dorsi. Together, this study provides evidence that herbal tea residues can be used as a functional roughage to modulate rumen fatty acid composition and muscle glucolipid metabolism.

Six papers in this collection aimed to develop effective methane mitigation strategies in ruminants. Brede et al. used the rumen simulation technique (RUSITEC) in a 38-day trial to investigate the effect of a feed additive consisting of garlic powder and bitter orange extracts on rumen fermentation and methane production. This feed additive does not appear to affect the microbial or prokaryote population but does alter ruminal SCFA concentration profiles. The intervention led to a transient reduction in the abundance of *Methanomicrobia*, the most abundant archaeal group in the system, and a concomitant reduction in the copy number of a key gene in methanogenesis that encodes methyl coenzyme-M reductase, resulting in a transient inhibition in methane production. However, the practical impact of the feed additive on production settings remained to be documented. Künzel et al. used a similar RUSITEC system to understand how two brown seaweeds from Iceland affect methanogenesis and nutrient conversion. Their findings demonstrated that while the inclusion of two dose levels of both seaweeds tested shows a small yet significant reduction in methane production, the intervention also resulted in a concomitant reduction on overall rumen fermentation, which raised serious questions of their utilization by farmers. Bharanidharan et al. also evaluated the potential of seeds of *Pharbitis nil*, a plant species widely distributed in East Asia, as a possible dietary strategy for ruminal methane mitigation using *in vitro* and *in sacco* methods. The plant indeed inhibited methanogenesis by up to 50%, depending on the dose tested. Furthermore, this plant can also modulate the rumen microbiota, including decreasing the Bacteroidetes-to-Firmicutes ratio. The additional benefit of this plant as feed additive lies in its antiprotozoal potential. In addition to the *in vitro* studies, Thirumalaisamy et al. examined the effect of a long-term supplementation of silkworm pupae oil on methane emissions and production traits in adult sheep. A daily silkworm pupae oil supplementation for 180 days reduced methane emissions up to 25% while maintaining higher bodyweight gains. However, the study also confirmed the transient nature of silkworm pupae oil on methane inhibition where daily methane emissions reverted to pre-supplementation levels shortly after the supplementation ceased. Similarly, methane emissions can be decreased by up to 90% in calves receiving a combination of two known methane inhibitors, chloroform and 9,10-anthraquinone, in the first 12 weeks of life (Cristobal-Carballo et al.), compared to control calves. Moreover, no negative impacts of the treatment on dry matter intake and growth were noticed. Similar to the *in vitro* data, these methanogen inhibitors have limited effect on rumen bacterial communities while reducing the abundance of methanogens, such as *Methanobrevibacter* and *Methanosphaera*. However, once methane inhibition ceased, the methanogen community, rumen metabolites and hydrogen emissions reversed to the baseline, providing further evidence that imprinting a rumen microbiota with lower methane yield during early life is still difficult.

To overcome the difficulties of transient methanogenesis inhibition of known methane inhibitors and natural products for on-farm pragmatic applications, Smith et al. took a different approach, aiming to develop strategies for genetic selection to achieve permanent and accumulative reductions to the methane emission related to livestock farming. Using residual methane emissions (RME) as an optimal phenotype for assessing the methanogenic potential of ruminants, they measured RME for 282 crossbred finishing beef cattle and then ranked the animals with high or low RME, differing in ~30% difference in RME. The rumen in the low-RME cattle tends to have higher abundances of lactic-acid-producing bacteria (LAB) as well as significantly higher abundances of methanogens, such as *Methanosphaera*, the latter of which is likely under the control of host genetics and negatively correlated with RME and positively correlated with rumen propionate levels. These taxa can be used as potential biomarkers of the methanogenic potential in beef cattle. The findings from this study likely generated microbial targets for future environmentally focused breeding programs.

The valuable contribution of this special collection to the rumen microbiology literature is vividly evident by its initial impacts, a total of 6,367 downloads and 30,255 views during the first few months of its inception. The collection was ranked one of Outstanding Research Topics of 2022 by Frontiers. The findings from these studies provide novel insights into host-rumen microbe interactions, which should facilitate the development and optimization of ruminal manipulation and enteric methane control strategies. We wish to express our gratitude to all contributing authors for their outstanding research efforts. Without their hard work, this collection would not have come into existence. Finally, we want to thank the editorial board members and staff of Frontiers in Microbiology, particularly the section editors of Systems Microbiology, for their support to this Research Topic.

## Author contributions

RL: conceptualization, data curation, and writing—original draft preparation. CM and DM: writing—revision. All authors contributed to the article and approved the submitted version.

